# Burden of neck pain and associated factors among smart phone user students in University of Gondar, Ethiopia

**DOI:** 10.1371/journal.pone.0256794

**Published:** 2021-09-07

**Authors:** Sileshi Ayhualem, Abayneh Alamer, Sisay Deme Dabi, Kefale Getie Bogale, Abey Bekele Abebe, Mulugeta Bayisa Chala

**Affiliations:** 1 Department of Human Anatomy, School of Medicine, College of Medicine and Health Science, University of Gondar, Gondar, Ethiopia; 2 Department of Physiotherapy, School of Medicine, College of Medicine and Health Science, Mekelle University, Mek’ele, Ethiopia; 3 School of Rehabilitation Therapy, Queen’s University, Kingston, Canada; 4 Department of Physiotherapy, School of Medicine, College of Medicine and Health Science, University of Gondar, Gondar, Ethiopia; Universiti Kebangsaan Malaysia, MALAYSIA

## Abstract

**Background:**

Mobile technology has spread rapidly around the globe. In 2018 the numbers of mobile subscribers in Ethiopia hit 66.2 million. Musculoskeletal complaints related to smartphone use in different body parts have been reported ranging from 8.2% to 89.9%. Neck pain has the highest prevalence rate, which ranges from 17.3% to 67.8%. However, there is limited evidence on the burden of neck pain related to Smartphone usage and no research is done in Ethiopia. Therefore, this study was conducted to determine the burden of neck pain and factors associated with smartphone use in Ethiopia.

**Purpose:**

The objective of this study was to identify the prevalence and factors associated with neck pain among smartphone users at University of Gondar.

**Methods:**

Institutional based cross-sectional study was conducted from November to December 2019 to determine the prevalence and associated factors of neck pain, with a sample of 845 university student smartphone users at University of Gondar, Ethiopia. A self-administered questionnaire adapted from the Nordic musculoskeletal questionnaire was used to collect data. Independent variables which had a significant association were identified using logistic regression models. Results were reported by using texts and frequency distribution tables.

**Results:**

Out of 845 questionnaires distributed, 808 students responded; hence, the response rate was 95.6%. The overall prevalence of neck pain among smart phone users in the past 12 months was 47.4% (95% CI, 44.1–50.9%). Attending 5^th^ year (AOR: 3.907, 95% CI: 1. 952–7.82) and 6^th^ year (AOR: 2.93,95% CI: 1,304–6.59), regular physical exercise (AOR: 2.405, 95% CI: 1.549–3.734), cigarette smoking (AOR: 5.415, 95% CI: 2.685–10.919), residency (AOR: 1.681, 95% CI: 1.181–2.391), break while using smartphone (AOR: 3.253 95% CI: 2.252–4.699), used smartphone > 6 hour per day (AOR: 2.782 (1.528 95% CI: 1.528–5.063), used other devises (AOR: 3.158 95% CI: 2.128–4.689), number of social media used daily (AOR: 2.007 95% CI: 1.228–3.2788), used devise for playing game (AOR: 1.484 95% CI: 1.024–2.15) were factors significantly associated with neck pain.

**Conclusion:**

The current study depicted that nearly half of the study participants reported neck pain in the past 12 months. Attending last year of university, personal characteristics, use of smart phone for longer period, playing game, not taking break, other electronic device use, increased number of social media use were associated with neck pain among smartphone users.

## Introduction

Mobile technology has spread rapidly around the globe. Today, it is estimated that more than 5 billion people have mobile devices, and over half of these communication devise are smart phones [1]. Young adults today have grown up with smartphones as an evident part of their lives. As the use of electronic devise has become more significant nowadays due to the various functions it offers to the users, it has been reported that there is an increase in ownership and usage of electronic devises among young adults [1–4]. In a recent study smart phone ownership among adults aged 18 to 34 was reported as 92% and 95% n the USA and Australia respectively [4]. In 2016, 26% and 28% of young adults in Kenya and Nigeria respectively owned smartphones [3]. However, the proportion increased to 41% in Kenya and 39% Nigeria in 2019 [1]. In 2016 it is reported that 4% of young adults in Ethiopia owned smartphones and 41% of adult internet users accessed internet at least once a day [3]. Within nearly every country, in both advanced economies and emerging and developing nations, those people in the 18–34 age range are much more likely to be internet and smart phone users compared with those aged 35 and older. It is also indicated that younger internet users also tend to access the internet at least once a day and participate in social networking at higher rates than their older counterparts [3–5]. Previous studies have reported high prevalence of musculoskeletal pain among university students related to electronic devise use. Musculoskeletal complaints related to smart phone use in different body parts have been reported ranging from 8.2% to 89.9% [6]. A study conducted in Canada showed that 84% of students who used smartphone reported musculoskeletal pain in at least one body part and among them 52% reported pain in the right shoulder, 46% in the left shoulder, 68% in the neck and 62% in the upper back [7]. Another studies conducted in different country reported neck and upper extremity pain as follows: 71.2% in Saudi Arabia [8], 20.1% in Malaysia [9], and 19.75%-32.50% in Thailand [10]. The annual prevalence of musculoskeletal pain related to computer use in African population is as follows: 30–64% in Nigeria [11], 30–64% in Sudan [12], and 5.3–38.95% in Kenya [13].

Musculoskeletal pain has also been recognized as a source of significant pain, disability and disadvantage for the injured person and a substantial burden on millions of people in both developed and developing countries. It affects all age groups and can also have a major impact on worker function, performance and productivity [14]. More over; neck, shoulder and upper extremity pain has pain reported as the major cause of sickness, reduced educational attainment and truancy from university lesson [15].

Studies show that forward head posture adopted while using electronic devise has been identified as one of the risk factors for musculoskeletal pain [7–9, 11, 16]. It is also indicated that increased neck flexion angle while using electronic devise is one of the risk factors for musculoskeletal pain [16–18]. Flexing the head forward at varying degrees increased weight loads on cervical spine dramatically and this increased stress potentially leads to early wear, tear, degeneration, and possibly surgery [19].

Different factors including socio-demographic factors like gender, age, body mass index (BMI); behavioral and personal factors like smoking, drinking habit, physical exercise; electronic characteristics such as type of electronic device, time spent per day, activities like texting, browsing internet and others factors such as adopted posture, duration of reading, academic year are considered as associated factors for musculoskeletal pain related to electronic device [20–22].

Even though the use of electronic device is on the rise and is associated with musculoskeletal symptoms, studies reported that there is limited evidence regarding electronic device use, and various aspects of its use (i.e. amount of usage, features, tasks and positions), and associated musculoskeletal symptoms and exposures [21]. Moreover, our extensive search showed that there are scarce published regional reports about prevalence of musculoskeletal pain in n the sub-Saharan region, yet we found none in Ethiopia. On the basis of those gaps we decided to assess the prevalence and associated factors of neck pain among smart phone users.

## Materials and methods

### Study design, area and period

Institutional based cross-sectional study was conducted from November to December 2019 at University of Gondar, Ethiopia. Gondar is located in the Amhara region, 738 km northwest of Addis Ababa; capital city of Ethiopia. University of Gondar, which has five campuses with a total of 32400 students during the study period, is located in Gondar town.

### Source and study population

All regular undergraduate students who were studying at University of Gondar were used as a source population and all sampled smart phone users from 2^nd^ to 6^th^ year of study who meet the inclusion criterion were the study participants.

### Inclusion criterion

Regular undergraduate students who used smartphones for one year and above were included in this study.

### Exclusion criterion

Students with previous history of musculoskeletal diseases, previous head and neck surgeries, previous diagnosis of cervical discs problems, and those with recent history of head and neck trauma were excluded from the study. We prepared a checklist with “Yes” or “No” questions prior to data collection to check those criteria. First year students were not included in the study because they were not enrolled at the university during data collection period.

### Sample size determination and sampling methods

Due to lack of similar studies in Ethiopia, Sample size was determined by the formula for single population proportions, using the assumption of a 5% level of significance, a marginal error of 5%, and 10% nonresponsive rate. The required sample size was obtained by the following calculation (n = sample size, p = prevalence, d = margin of error):

$$\text{n}={\text{z}}^{2}\;\text{p}\;\left(1-\text{p}\right)/{\text{d}}^{2},\;\text{n}={\left(1.96\right)}^{2}\text{x}\;\left(0.5\right)\;\left(0.5\right)/\;{\left(0.05\right)}^{2}=384.16=384.$$

Since the sampling technique was multistage, design effect 2 was used. The final sample size obtained was 845 by adding 10% non-response rate [23].

**Fig 1:**
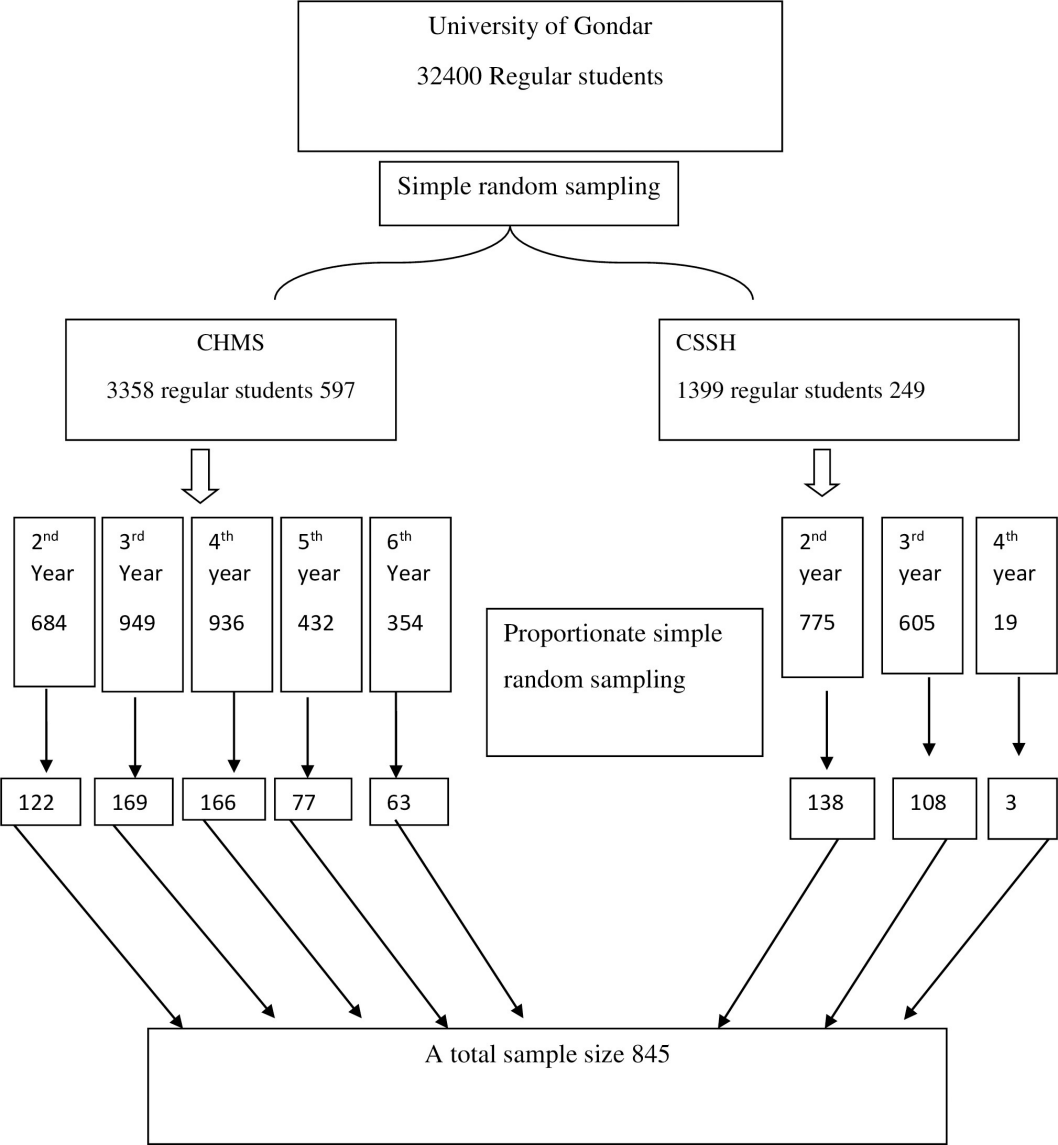


The University of Gondar has five campuses. Among these; College of Social Sciences and Humanities (CSSH) and College of Medicine and Health Sciences (CMHS) campuses were selected by simple random sampling. CHMS has 5 batches and 12 departments with a total of 3358 student and SHC has three batches and 17 departments with a total of 1399 students. The sample size needed from each college was proportionally allocated which was 597 for CHMS and 249 for CSSH. For each college the sample size needed from each batch is proportionally calculated. Each sample was selected by simple random sampling using the enrollment list obtained from the registrar office **(Fig 1)**.

### Study variables

Neck pain among smart phone users was the outcome variable while others like socio demographic characteristics, behavioral characteristics and pattern of smart phone use and postural characteristics while using smart phone are explanatory variables included in this study.

### Data collection procedures and quality control

The eligible students who fulfilled the inclusion criteria were included in the study after receiving an explanation about purpose of the study and taking informed written consent. Structured questionnaire adapted from standardized Nordic questionnaire [24] was used for the analysis of neck pain. The questionnaire included a body map to allow students to report neck pain by labeling the body location. Based on this questionnaire, neck pain for the last 12 months was asked by: “Have you at any time during the last 12 months had trouble (ache, pain or discomfort) in your neck?. The questionnaire was composed of three sections. The first part includes questions on socio-demographic data. The second part considered behavioral factors such as cigarette smoking, alcohol consumption and a habit of physical exercise. The last part included pattern of device use and postural characteristics such as purpose of smart phone use, total time per day usage of smart phone, number of social media use, break while using smart phone, frequent posture adopted while using smart phone, position of smart phone during use, style of holding smart phone and the occurrence of neck pain. Measuring tools like, portable weighting scale and tape meter were used. Data were collected by 5 trained physiotherapists, who were trained before the actual data collection concerning the approach, objective of the study and Ethical issues. The questionnaire was pre tested on 42 students at Bahirdar University, 184 km away from the study area, before a week of actual data collection, and some modifications were done based on the finding. The principal investigators and supervisors made a day- to- day on-site supervision during the whole period of data collection and checked each questionnaire daily for completeness and consistency. Ethical clearance was obtained from the Health Research Ethics Review Committee of University of Gondar. Written informed consent was obtained from the study participants after brief explanation about the objective, purpose, benefits, and risks of the study. Appropriate measures were taken to assure the confidentiality of information both during and after data collection.

### Operational definition

#### Neck pain

Neck pain is defined as pain, ache or discomfort in the area between the occipital and the first thoracic vertebra at any time in the last 12 months [25].

#### BMI: Body mass index (BMI)

weight in kilograms divided by the square of the height in meters (kg/m2). Underweight = BMI <18.50, Normal range = BMI b/n 18.50–24.99, Overweight = BMI between 25.00–29.99, Obese = BMI ≥30.00 [26].

#### Cigarette smoking

The individuals who reported cigarette smoking daily (at least one cigarette per day) or occasionally (less than one cigarette per day) were considered smoker [27].

#### Alcohol drinking

It was a consumption of any kind of alcohol by men and women at least twice a week for different purposes [28].

#### Physical exercise

Exercising any kinds of sport activity at least twice a week with duration of 30 minutes, or at least ≥150 minutes of moderate-intensity physical activity per week [29].

### Data management and analysis

The data was coded and checked before entry, then entered using Epi-info version 7.1, and after cleaning it was exported to Statistical Package for the Social Sciences (SPSS) Version 23 for analysis [30]. The results were presented using text, frequency distribution tables and percentages for descriptive statistics. Step wise binary logistic regression was used to identify factors associated with neck pain. Bivariate analysis was done to see the association between neck pain and independent variables. Variables with a P-value less than 0.2 were brought to multivariate analysis for controlling potential confounding factors [31]. The goodness of the model fit test was checked by the Hosmer–Lemeshow test (P value = 0.699), and the value suggested that the model is a good fit (p value > 0.05). Multicollinearity test was checked with a maximum variance inflation factor (VIF) = 3.8. The assumptions of each categorical variable having five and more than five cells were considered. Independent variables with a 95% confidence level and P-value less than 0.05 in the multivariate model were considered as statistically significant and presented with Adjusted Odds Ratio (AOR) with 95% CI.

## Results

### Socio demographic and behavioral characteristics of study participants

A total of 845 questionnaires were distributed to University of Gondar students, of which 808 Students responded; hence, the response rate was 95.6%. Majority of the respondents were male (57.9%). The mean age was 21.9 year (SD±2.15). More than half of the participants were orthodox Christians (56.6%) and grew up in urban areas (56.3%). A very large proportion (90.1%) had a normal body mass index (BMI). An insignificant minority of students (4.7%) was left-handed. Well over three quarter of students (80.7%) were not involved in regular exercise. A small proportion (11.1%) of the students were smokers and were alcohol drinkers (21.7%) **(Table 1)**.

**Table 1:** Socio demographic and behavioral characteristics of smart phone users at University of Gondar, 2020 (N = 808).

Variables	Frequency	Percent (%)
Sex		
Male	468	57.9%
Female	340	42.1%
Age		
≤20	229	28.3%
21–25	559	69.2%
≥26	20	2.5%
Religion		
Orthodox	455	56.3%
Muslim	148	18.3%
Protestant	191	23.6%
Other	14	1.7%
Place lived before joining university		
Urban	457	56.6%
Rural	351	43.4%
Marital status		
Single	789	97.65%
Married	19	1.35%
BMI		
Underweight	36	4.5%
Over weight	44	5.4%
Normal	728	90.1%
Handedness		
Right	770	95.3%
Left	38	4.7%
Physical Exercise		
Yes	156	19.3%
No	652	80.7%
Alcohol drinking		
Yes	175	21.7%
No	633	78.3%
Cigarette Smoking		
Yes	90	11.1%
No	718	88.9%

### Pattern of device use and postural characteristics of study participants

A large proportion of students used their smart phones daily for watching video (97.5%) and for reading (80.9%), while only 33.2% of the students used their smartphone for playing games daily. Surprisingly all participants used their smart phones daily for social media (100%). Majority of smartphone users (61.4%) have typical usage time of 3–6 hours per day. The most common posture during use of smart phone was sitting (56.3%) followed by standing (16%), lying on back (16%) and lying on chest (11.75%). When using smartphones 81.6% of students hold their phone below their eye level by adopting position of neck flexion. Over half (55.9%) of the students used both left and right hands to manipulate their smart phones. In addition, 62.7% of the students reported using other devices; 36.5% used laptop or tablet and 11% used desktop.

The majority of smart phone users (66.7%) used 2–3 different type of social media daily where as 16.8% of them used four or more type social media daily **(Table 2)**.

**Table 2:** Patterns of device use and postural characteristic of smart phone users at University of Gondar, 2020. (N = 808).

Variables	Frequency	Percent (%)
Use Smart Phone for reading		
Yes	654	80.9%
No	154	19.1%
Use smart phone for watching video		
Yes	788	97.5%
No	20	2.5%
Use smart phone for gaming		
Yes	268	33.2%
No	540	66.8%
Use smart phone for social media		
Yes	808	100%
No	0	0%
Total time usage phone per day		
<3	206	25.5%
3–6	496	61.4%
>6	106	13.1%
Break while using smart phone		
Take break	287	35.5%
No break	521	64.5%
Frequent posture adopted during the use Smart phone		
Sitting	455	56.3%
Standing	129	16%
Laying back	129	16%
Laying chest	95	11.75%
Position of phone		
At eye level	112	13.9%
Above eye level	37	4.6%
Below eye level	659	81.6%
Style of holding phone (hand in use)		
Right	351	43.4%
Left	5	0.6
Both	452	55.9
Use other devise		
Yes	507	62.7%
No	301	37.3%
Type of other devise use		
Laptop/tablet		
Yes	296	36.6%
No	512	63.4%
Desktop		
Yes	89	11%
No	719	89%
Number of social media		
One	133	16.5%
2–3	539	66.7%
>4	136	16.8%

### Prevalence of neck pain among smart phone users

Three hundred and eighty three (47.4% (95% CI, 44.1–50.9)) students reported experiencing neck pain in the past 12 months. Reported prevalence of neck pain was higher among female students (49.1%) as compared to male students (46.2%). The prevalence of neck pain was higher among 5^th^ (77.9%) and 6^th^ (78%) year students, students who were categorized as overweight (56.8%), students who came from urban areas (58.9%), students who are smoker(84.4%), students who used smartphones for gaming daily(56.3%), students who did not take break while using smartphone (57.6%), students who used smart phone for > 6 hours per day (64.2%), students who used ≥4 social media daily (61%) and students who used other electronic devices (58.4%).

The prevalence of neck pain was less among those who were involved in physical exercise (32.1%) and used only one social media daily (24.8%). Approximately same figure is reported among students who held their smartphones by one hand (47.5%) and by both hands (47.3%) while using it.

### Factors associated with neck pain among smart phone users

Variables that are potentially related to neck pain among smartphone users were identified: Sex, religion, year of study, BMI, residency, marital status, handedness, regular physical exercise, cigarette smoking, drinking alcohol, position of phone while using, frequent posture adopted while using smartphone, style of holding smartphone while using, break while using smartphone, total time per day smartphone usage, number of social media used daily, used smartphone for reading, used smartphone for gaming, used smartphone for watching video, usage of other electronic device.

Among those variables, sex, religion, posture adopted while using smart phone, style of holding smart phone while using, drinking alcohol and handedness were not significantly associated (p>0.2) with self reported neck pain among smartphone users in the bivariate logistic regression.

The remaining thirteen variables were evaluated and analyzed in the multivariate logistic regression. Finally, self reported neck pain among smart phone users was significantly associated (p<0.05) with regular physical exercise (AOR: 2.405, 95% CI: 1.549–3.734), Year of study; 5^th^ year (AOR: 3.907, 95% CI: 1.952–7.82) and 6^th^ year (AOR: 2.931 (1.304–6.59), cigarette smoking (AOR: 5.415, 95% CI: 2.685–10.919), residency (AOR: 1.681, 95% CI: 1.181–2.391), break while using smartphone (AOR: 3.253 95% CI: 2.252–4.699), used smart phone > 6 hours per day (AOR: 2.782 (1.528 95% CI: 1.528–5.063), used other devices (AOR: 3.158 95% CI: 2.128–4.689), number of social media used daily (AOR: 2.007 95% CI: 1.228–3.2788), used device for playing game (AOR: 1.484 95% CI: 1.024–2.15) **Table 3**.

**Table 3:** Bivariate and multivariate logistic regression analysis of associated factors with neck pain smart phone user students, 2020. (N = 808).

Variables	Neck pain Yes	COR (95% CI)	P- value	AOR (995% CI)	P-value
Year of study					
2^nd^ year	102(43.4%)	1			
3^rd^ year	111(41.1%)	0.91(0.639–1.297)	0.603		
4^th^ year	64(38.3%)	0.81(0.54–1.214)	0.308		
5^th^ year	60(77.9%)	**4.602(2.533–8.361)** *	<0.001	**3.907(1.952–7.82)** **	<0.001
6^th^ year	46(78%)	**4.614(2.367–8.994)** *	<0.001	**2.931(1.304–6.59)** **	<0.001
Regular physical exercise					
Yes	50(32.1%)	1			
No	333(51.1%)	**2.213(1.529–3.203)** *	<0.001	2.405(1.549–3.734) **	<0.001
Cigarette smoking					
Yes	76(84.4%)	7.268(4.033–13.096) *	<0.001	**5.415(2.685–10.919)** **	<0.001
No	306(42.8%)	1			
Residency					
Urban	269(58.9%)	**2.975(2.224–3.974)** *	<0.001	**1.681(1.181–2.391)** **	0.004
Rural	114(32.5%)	1			
BMI					
Underweight	10(27.8%)	-**0.42(0.2–0.883)** *	0.022		
Over weight	25(56.8%)	1.437(0.778–2.655)	0.247		
Normal	348(47.8%)	1			
Take break while using					
Take break	83(28.9%)	1			
No break	300(57.6%)	**3.336(2.45–4.543)** *	<0.001	**3.253(2.252–4.699)** **	<0.001
Position of smart phone while using					
At eye level	45(40.2%)	1			
Above eye level	14(37.8%)	-0.906(0.422–1.946)	0.801		
Below eye level	324(49.2%)\	1.44(0.958–2.164)	0.079		
Total time smart phone usage per day					
<3 hour	92(44.7%)	1			
3–6 hour	223(45%)	1.012(0.73–1.403)	0.942		
>6 hour	68(64.2%)	**2.217(1.368–3.593)** *	0.001	**2.782(1.528–5.063)** **	0.001
Number of social media					
One	33(24.8%)	1			
2–3	267(49.5%)	**2.975(1.939–4.564)** *	<0.001	**2.007(1.228–3.278)** **	0.005
>3	83(61%)	**4.746(2.813–8.007)** *	<0.001	**2.546(1.36–4.764)** **	0.003
Use smart phone for gaming					
Yes	151(56.3%)	**1.713(1.275–2.302)** *	<0.001	**1.484(1.024–2.15)** **	0.037
No	237(43%)	1			
Use smart phone for watching video					
Yes	377(47.8%)	2.14(0.814–5.626)	0.123		
No	6(30%)	1			
Use smart phone for reading					
Yes	327(50%)	**1.75(1.218–2.514)** *	0.002		
No	56(36.4%)	1			
Use other electronic devise					
Yes	296(58.4%)	**3.451(2.542–4.683)** *	<0.001	**3.158(2.128–4.687)** **	<0.001
No	87(28.9%)	1			

* = significant association (bivariate),

** = significant association (multivariate), COR = crude odds ratio, AOR = adjusted odds ratio, 1.00 = references, * = p-value < 0.05, [95% CI:95% Confidence Interval].

## Discussion

This is the first study that investigated the prevalence and associated factor of neck pain among smart phone users in Ethiopia. The prevalence of neck pain among smartphone users in the past 12 month was 47.4% (95% CI, 44.1–50.9%). The result indicates that neck pain is common among smart phone users. This finding is pursuant to a study conducted in India (46.9%) [32]. However, other studies on the prevalence of neck pain among smartphone users reported higher or lower numbers. The prevalence of neck pain in our study is lower than studies conducted in China (72.9%) [33], Saudi Arabia (71.2%) [8], Brazil (66.7%) [34], Singapore (74%) [35], and Taiwan (52%) [36]. The disparity observed in the prevalence of neck pain could be due to the difference in the study area, sample size, sampling method, and inclusion criterion and data collection procedure. What is more, the disparity could also be due to factors such as facility provided for the students at their institution, exposure to electronic device, pattern of use, daily usage in hours and other environmental factors between Ethiopia and those mentioned study areas; these a fore mentioned play a major role for the observed prevalence rate. The study done in China used convenient sampling method with small sample size and included students who had used various types of electronic device [33]. The study done in Saudi Arabia used very small sample size and recruited only medical students [8]. The study conducted in Brazil used fairly large sample size; however, the sampling method was different stratified based on the course they took[]. A **s**tudy from Singapore included primary and secondary school students who used tablets and smartphones and implemented a longitudinal prospective study. Moreover, distribution of musculoskeletal symptoms in body regions-neck and shoulder-were examined in combination [35]. The sample size of the study done in Taiwan was small and its focus was grade 2–5 students, yet as to sampling, it used convenient sampling [36]. However, our study has employed cross-sectional study and multi stage sampling technique stratified by academic years with fairly large sample size, and included students who used smartphones for one or more years from different colleges and different departments. In addition, self- administered questionnaires were used for data collection, and distributions of musculoskeletal symptoms in neck region were examined.

On the other hand, the prevalence rate in our finding shows higher figures than studies done in Malaysia (18%) [9] and Thailand (32.5%) [37]. The difference in sampling technique, sample size and inclusion criterion happen to be the cause of the disparity. The studies in Thailand and Malaysia had very small sample size and the study in Thailand used cluster sampling and included smart phone users who had used smartphones for less than one year [9, 37]. However, what makes our study different is that we have used multi stage sapling technique, large sample size and recruited smart phone users who had used for one year or more.

In this study 5^th^ and 6^th^ year of study were 3.907 and 2.93 times more likely to develop neck pain, respectively. This result is supported by studies done China [38]. Prolonged use of electronic device, long clinical practice hour, use of different social media platforms, usage of smart phone for longer years and exposure to psychosocial hazards might have contributed to such a result be due to, which imply that neck pain might develop over the course of the study.

Our study showed students who were not engaged in regular physical exercise were 2,405 times more likely to develop neck pain than the ones who did physical exercise regularly. The result was consistent with a study done in China [39]. This might be due to the fact that less explained in short; less flexible and weak muscles can cause neck pain as they can cause misalignment of neck anatomical structures. In contrast, students who did regular physical exercise were able to strengthen, lengthen, improve flexibility and make their muscles and ligaments strong to support and keep the neck alignment for proper functioning and preventing injury [29].

This study also depicted that students who were smokers are 5.415 times more likely to develop neck pain than non smokers, which is in line with a study done in Thailand [37].

Our study revealed that students whose upbringing is in urban areas were 1.681 times more likely to develop neck pain than those students who grew up in rural area. This finding is justified by the assumption that students from urban areas utilize electronic devices more than students from rural areas, for students from urban areas might have exposure and access to new technologies.

Students who did not take break while using smartphones were 3.253 times more likely to develop neck pain than their counter parts. This result could be because of the negative effect of prolonged use of smartphones on posture which could increase stress on muscle, ligaments and tendons [40]. The study also showed that long time use of smart phone has significantly associated with neck pain (P< 0.05) [41].

In our study students who used smart phone for six or more hours per day are 2.782 times more likely to develop neck pain. This finding is supported by a study done elsewhere [11, 20, 34, 36, 39, 42]. The finding implies that prolonged use of smart phone is likely to increase the risk of neck pain.

Students who used two to three and three or more types of social media per day were 2.007 and 2.546 times more likely to develop neck pain, respectively. This finding is consistent with previous studies done elsewhere [35, 43]. Such result might be associated with the increased hour in using different social media platforms and they do that nonstop.

In our study students who used their smart phones for playing game daily were 1.484 times more likely to develop neck pain than their counter parts. A report of a study done in South Africa recorded similar results [44]. This finding advocates that active tasks like gaming were significantly associated with higher head and neck flexion angle and increased muscle activities of upper trapezius, deltoid and cervical extensors [45]. Therefore, playing game daily could increase stress on the neck musculatures and could result in neck pain.

Students who used not only smartphones but also other electronic devices were 3,158 times more likely to develop neck pain than those students who used only smart phone. This finding is in line with a similar study done in Singapore, which reported that those who use varied communication devices, other than smartphones, were 1.61 times more likely to develop neck pain [35]. This finding suggests that as students use more number of devices daily, they happen to spend more hours per day, which in turn could increase the risk of developing neck pain.

## Conclusion

The current study depicted that nearly half of the study participants reported neck pain in the past 12 months. Attending last year of university, cigarette smoking, urban residency, not involving in regular physical exercise, daily use of smart phone for longer period, playing game, not taking break while using smart phone, using other electronic device, increased number of social media use were associated with neck pain among smart phone users. Students should be aware regarding wise use of smart phones and other electronic device. Regular break while using smart phone could be important to reduce the risk of developing neck pain. Moreover, universities should promote regular physical exercise among students by building facilities needed for this purpose. In a nutshell more attention should be given towards increasing awareness regarding appropriate use of mobile phone in order to decrease prevalence of neck pain among university smart phone user students. This study could not determine the severity of neck pain, alongside with its duration, and nature of pain among the study participants. Thus, this finding warrant for further study on neck pain severity and its precipitating factors among smart phone user students.

## Supporting information

S1 Questionnaire English version of the research questionnaire. (DOCX)

## Abbreviations

AOR: Adjusted odds ratio
BMI: Body mass index
CHMS: College of medicine and health science
CI: Confidence interval
OR: Odds ratio
SHC: Social and Humanity College
SPSS: Statistical package for social science
USA: United state of America
VIF: variables inflation factors

## References

[1] Taylor K, Silver L. Smartphone ownership is growing rapidly around the world, but not always equally. Pew Research Center. 2019;5.

[2] Liadi OF. College students and smartphone ownership: symbolic meanings and smartphone consumption among Nigerian Students. Acta Universitatis Danubius Communicatio. 2016;10(1):17–31.

[3] Poushter J. Smartphone ownership and internet usage continues to climb in emerging economies. Pew research center. 2016;22(1):1–44.

[4] Rideout VJ, Foehr UG, Roberts DF. Generation M 2: Media in the Lives of 8-to 18-Year-Olds. Henry J Kaiser Family Foundation. 2010.

[5] Gustafsson E, Thomée S, Grimby-Ekman A, Hagberg M. Texting on mobile phones and musculoskeletal disorders in young adults: a five-year cohort study. Applied ergonomics. 2017;58:208–14. doi: 10.1016/j.apergo.2016.06.012 27633215

[6] Zirek E, Mustafaoglu R, Yasaci Z, Griffiths MD. A systematic review of musculoskeletal disorders related to mobile phone usage. Musculoskeletal Science and Practice. 2020:102196. 10.1016/j.msksp.2020.102196 32861360

[7] Berolo S, Wells RP, Amick III BC. Musculoskeletal symptoms among mobile hand-held device users and their relationship to device use: a preliminary study in a Canadian university population. Applied ergonomics. 2011;42(2):371–8. doi: 10.1016/j.apergo.2010.08.010 20833387

[8] AlZarea BK, Patil SR. Mobile phone head and neck pain syndrome: proposal of a new entity. Headache. 2015;251:63.

[9] Kalirathinam D, Manoharlal MA, Mei C, Ling CK, Sheng TWY, Jerome A, et al. Association between the usage of Smartphone as the risk factor for the prevalence of upper extremity and neck symptoms among University students: A cross-sectional survey based study. Research Journal of Pharmacy and Technology. 2017;10(4):1184–90.

[10] Vate-U-Lan P. Text neck epidemic: a growing problem for smart phone users in Thailand. International Journal of the Computer, the Internet and Management. 2015;23(3):551–6.

[11] Ayanniyi O, Ukpai B, Adeniyi A. Differences in prevalence of self-reported musculoskeletal symptoms among computer and non-computer users in a Nigerian population: a cross-sectional study. BMC Musculoskeletal Disorders. 2010;11(1):177. 20691092 10.1186/1471-2474-11-177 PMC2927507

[12] Eltayeb SM, Staal JB, Hassan AA, Awad SS, de Bie RA. Complaints of the arm, neck and shoulder among computer office workers in Sudan: a prevalence study with validation of an Arabic risk factors questionnaire. Environmental Health. 2008;7(1):33. doi: 10.1186/1476-069X-7-33 18588691 PMC2474607

[13] Omondi S, editor Risk Factors for upper body musculoskeletal discomforts among computer users in Kenya. Scientific Conference Proceedings; 2014.

[14] Hammerschmidt DM. The prevalence of work-related musculoskeletal disorders in certified members of the National Athletic Trainers’ Association: North Dakota State University; 2008.

[15] Rose K. The effect of neck pain and headaches on the academic performance of college students. JNMS-JOURNAL OF THE NEUROMUSCULOSKELETAL SYSTEM. 2000;8(4):118–23.

[16] Lee H-j. Neck pain and functioning in daily activities associated with smartphone usage. The Journal of Korean Physical Therapy. 2016;28(3):183–8.

[17] Guan X, Fan G, Wu X, Zeng Y, Su H, Gu G, et al. Photographic measurement of head and cervical posture when viewing mobile phone: a pilot study. European Spine Journal. 2015;24(12):2892–8. doi: 10.1007/s00586-015-4143-3 26206292

[18] Haneen M. Effects of texting on neck muscle activity and neck flexion in college students. 2018.

[19] Hansraj KK. Assessment of stresses in the cervical spine caused by posture and position of the head. Surg Technol Int. 2014;25(25):277–9. 25393825

[20] Xie Y, Szeto G, Dai J. Prevalence and risk factors associated with musculoskeletal complaints among users of mobile handheld devices: A systematic review. Applied ergonomics. 2017;59:132–42. doi: 10.1016/j.apergo.2016.08.020 27890121

[21] Toh SH, Coenen P, Howie EK, Straker LM. The associations of mobile touch screen device use with musculoskeletal symptoms and exposures: A systematic review. PloS one. 2017;12(8):e0181220. doi: 10.1371/journal.pone.0181220 28787453 PMC5546699

[22] Saueressig IB, Oliveira VMAd, Xavier MKA, Santos LRAd, Silva KMA, Araújo RCd. Prevalence of musculoskeletal pain in adolescents and its association with the use of electronic devices. Revista Dor. 2015;16(2):129–35.

[23] Naing L, Winn T, Rusli B. Practical issues in calculating the sample size for prevalence studies. Archives of orofacial Sciences. 2006;1:9–14.

[24] Kuorinka I, Jonsson B, Kilbom A, Vinterberg H, Biering-Sørensen F, Andersson G, et al. Standardised Nordic questionnaires for the analysis of musculoskeletal symptoms. Applied ergonomics. 1987;18(3):233–7. doi: 10.1016/0003-6870(87)90010-x 15676628

[25] Fejer R, Hartvigsen J. Neck pain and disability due to neck pain: what is the relation? European Spine Journal. 2008;17(1):80–8. doi: 10.1007/s00586-007-0521-9 17955268 PMC2365525

[26] Organization WH. Obesity: preventing and managing the global epidemic: World Health Organization; 2000. 11234459

[27] Khader Y, Alsadi A. Smoking habits among university students in Jordan: prevalence and associated factors. EMHJ-Eastern Mediterranean Health Journal, 14 (4), 897–904, 2008. 2008. 19166173

[28] White I, Altmann D, Nanchahal K. ’Optimal’levels of alcohol consumption for men and women at different ages. 2003. 10.1136/bmj.325.7357.191 PMC117446 12142306

[29] Tsauo J-Y, Lee H-Y, Hsu J-H, Chen C-Y, Chen C-J. Physical exercise and health education for neck and shoulder complaints among sedentary workers. Journal of Rehabilitation Medicine. 2004;36(6):253–7. doi: 10.1080/16501970410029807 15841601

[30] George D, Mallery M. IBM SPSS Sta s cs 23 Step by Step A Simple Guide and Reference (14th Edi on). Boston, MA: Allyn and Bacon; 2016.

[31] In Lee K, Koval JJ. Determination of the best significance level in forward stepwise logistic regression. Communications in Statistics-Simulation and Computation. 1997;26(2):559–75.

[32] Ahmed S, Akter R, Pokhrel N, Samuel AJ. Prevalence of text neck syndrome and SMS thumb among smartphone users in college-going students: a cross-sectional survey study. Journal of Public Health. 2019:1–6. doi: 10.1093/pubmed/fdz027 30973958

[33] Woo EH, White P, Lai CW. Musculoskeletal impact of the use of various types of electronic devices on university students in Hong Kong: An evaluation by means of self-reported questionnaire. Manual therapy. 2016;26:47–53. doi: 10.1016/j.math.2016.07.004 27479091

[34] De Vitta A, Candido JP, Bento T, Cornelio GP, de Oliveira Perrucini P, Fernandes JAA, et al. Neck pain and factors associated in University Students: a cross sectional study. Ciência em Movimento.22(43):89–101.

[35] Toh SH, Coenen P, Howie EK, Smith AJ, Mukherjee S, Mackey DA, et al. A prospective longitudinal study of mobile touch screen device use and musculoskeletal symptoms and visual health in adolescents. Applied Ergonomics. 2020;85:103028. doi: 10.1016/j.apergo.2019.103028 32174368

[36] Yang S-Y, Chen M-D, Huang Y-C, Lin C-Y, Chang J-H. Association between smartphone use and musculoskeletal discomfort in adolescent students. Journal of community health. 2017;42(3):423–30. doi: 10.1007/s10900-016-0271-x 27734246

[37] Namwongsa S, Puntumetakul R, Neubert MS, Boucaut R. Factors associated with neck disorders among university student smartphone users. Work. 2018;61(3):367–78. doi: 10.3233/WOR-182819 30373996

[38] Chan LLY, Wong AYL, Wang MH, Cheung K, Samartzis D. The prevalence of neck pain and associated risk factors among undergraduate students: A large-scale cross-sectional study. International Journal of Industrial Ergonomics. 2020;76:102934.

[39] Shan Z, Deng G, Li J, Li Y, Zhang Y, Zhao Q. Correlational analysis of neck/shoulder pain and low back pain with the use of digital products, physical activity and psychological status among adolescents in Shanghai. Plos one. 2013;8(10):e78109. doi: 10.1371/journal.pone.0078109 24147114 PMC3795657

[40] Jung SI, Lee NK, Kang KW, Kim K, Do YL. The effect of smartphone usage time on posture and respiratory function. Journal of physical therapy science. 2016;28(1):186–9. doi: 10.1589/jpts.28.186 26957754 PMC4756000

[41] Lee JI, Song HS. The correlation analysis between hours of smartphone use and neck pain in the Gachon university students. The Acupuncture. 2014;31(2):99–109.

[42] Blair B, Gama M, Toberman M. Prevalence and risk factors for neck and shoulder musculoskeletal symptoms in users of touch-screen tablet computers. 2015.

[43] Al-Dubai SAR, Ganasegeran K, Al-Shagga MAM, Yadav H, Arokiasamy JT. Adverse health effects and unhealthy behaviors among medical students using Facebook. The scientific world journal. 2013;2013. doi: 10.1155/2013/465161 24453859 PMC3888741

[44] Goon D. Musculoskeletal problems associated with university students computer users: a cross-sectional study. Online Journal of Health and Allied Sciences. 2017;16(2).

[45] Al-Hadidi F, Bsisu I, AlRyalat SA, Al-Zu’bi B, Bsisu R, Hamdan M, et al. Association between mobile phone use and neck pain in university students: A cross-sectional study using numeric rating scale for evaluation of neck pain. PloS one. 2019;14(5):e0217231. doi: 10.1371/journal.pone.0217231 31107910 PMC6527223

