# Supplementary material for: Burden of neck pain and associated factors among smart phone user students in University of Gondar, Ethiopia
Source: PLoS One. 2021 Sep 7;16(9):e0256794. doi: 10.1371/journal.pone.0256794 (PMC8423292; doi:10.1371/journal.pone.0256794)
Supplement: S1 Questionnaire — (DOCX) [file pone.0256794.s001.docx]

**English version questionnaire**

**Survey information**

1. Date of data collection ……………………….
2. Time of interview (24 hour clock)…………….

Please answer by putting cross in the appropriate box or circle the possible answer and write a correct response on blank space for those questions which needs specification and skip to indicated question if you respond “No” for those questions.

Demographic data

1. Sex Male Female
2. Age…………………
3. Academic year………………………………………………………………………
4. Place lived before joining university
5. Urban
6. Rural
7. Religion
8. Orthodox
9. Muslim
10. Protestant
11. Other
12. Marital status
13. Single
14. Married
15. Handedness
16. Right handed
17. Left handed

**Physical measurements**

1. Height in cm (objectively)…………………
2. Weight in Kg (objectively)…………………
3. BMI…………………………

**Personal characteristics**

1. Habit of smoking
2. Yes
3. No
4. Habit of alcohol drinking
5. Yes
6. No
7. Habit of physical exercise
8. Yes
9. No

**II characteristics related to Smartphone devise usage**

1. Do you use your Smartphone daily for reading?
2. Yes B. No
3. Do you use your Smartphone daily for playing game?
4. Yes B. No
5. Do you use your Smartphone daily for texting or chatting or for other purpose on social media?
6. Yes B. No
7. How many social media (like face book, telegram, what’s app, imo etc.) do you use in a typical day.
8. One
9. Two
10. Three
11. Four
12. Five or more
13. Do you use your Smartphone daily for watching video?
14. Yes B. No
15. How many hours do you spend on typical day on your Smartphone for texting and chat, gaming, watching video, reading, and other activities?
16. Total time usage per day………………………..(hr) or ………………(min)
17. While you are using the Smartphone for the above activities (such us chatting, gaming, watching video, reading etc.) do you take a break?
18. No break
19. Take break
20. Have you been using other electronic devise like tablet, laptop or desktop or others for playing game, chatting, reading, watching video, browsing internet or other activities in addition to Smartphone?
21. Yes
22. No
23. Do you use laptop/tablet?
24. Yes
25. No
26. Do you use desktop?
27. Yes
28. No

**IV Related to posture**

1. How do you hold your Smartphone while you are using it?
2. Below eye level
3. At eyelevel
4. Above eye level
5. How is your style of holding (hand in use) your Smartphone while you are using it?
6. Using right hand only
7. Using your left hand only
8. Using both hands
9. What is the frequent posture you adopted during the use of Smartphone?
10. Sitting
11. Standing
12. Laying on back
13. Laying on chest

**Characteristics related to pain**

1. Have you at any time during the last 12 month had trouble (pain, discomfort, and ache) at your neck?
2. Yes
3. No

Figuer 1: The diagram below shows the approximate position of body part referred to neck.

Please concentrate on the shaded area, ignore any trouble you may have in the adjacent part of the body.


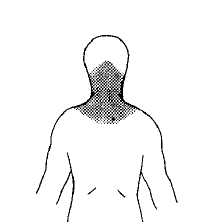


**Neck pain**

Kuorinka I ..et al 1987
